# Exploring Valine Metabolism in Astrocytic and Liver Cells: Lesson from Clinical Observation in TBI Patients for Nutritional Intervention

**DOI:** 10.3390/biomedicines8110487

**Published:** 2020-11-10

**Authors:** Sarah Sonnay, Nicolas Christinat, Jonathan Thevenet, Andreas Wiederkehr, Anirikh Chakrabarti, Mojgan Masoodi

**Affiliations:** 1 Lipid metabolism, Nestlé Research, Nestlé Institute of Health Sciences,1015 Lausanne, Switzerland; sarah.sonnay@bluewin.ch (S.S.); Nicolas.Christinat@rd.nestle.com (N.C.); Anirik@gmail.com (A.C.); 2 Mitochondrial Function, Nestlé Research, Nestlé Institute of Health Sciences, 1015 Lausanne, Switzerland; Jonathan.Thevenet@rd.nestle.com (J.T.); andreas.wiederkehr@rd.nestle.com (A.W.); 3 Institute of Clinical Chemistry, Inselspital, Bern University Hospital, 3010 Bern, Switzerland

**Keywords:** valine, β-hydroxyisobutyrate, 2-ketoisovaleric acid, traumatic brain injury, liver

## Abstract

The utilization of alternative energy substrates to glucose could be beneficial in traumatic brain injury (TBI). Recent clinical data obtained in TBI patients reported valine, β-hydroxyisobutyrate (ibHB) and 2-ketoisovaleric acid (2-KIV) as three of the main predictors of TBI outcome. In particular, higher levels of ibHB, 2-KIV, and valine in cerebral microdialysis (CMD) were associated with better clinical outcome. In this study, we investigate the correlations between circulating and CMD levels of these metabolites. We hypothesized that the liver can metabolize valine and provide a significant amount of intermediate metabolites, which can be further metabolized in the brain. We aimed to assess the metabolism of valine in human-induced pluripotent stem cell (iPSC)-derived astrocytes and HepG2 cells using ^13^C-labeled substrate to investigate potential avenues for increasing the levels of downstream metabolites of valine via valine supplementation. We observed that 94 ± 12% and 84 ± 16% of ibHB, and 94 ± 12% and 87 ± 15% of 2-KIV, in the medium of HepG2 cells and in iPSC-derived astrocytes, respectively, came directly from valine. Overall, these findings suggest that both ibHB and 2-KIV are produced from valine to a large extent in both cell types, which could be of interest in the design of optimal nutritional interventions aiming at stimulating valine metabolism.

## 1. Introduction

Traumatic brain injury (TBI) is associated with high economic costs and clinical burden. It is characterized by abnormal brain energy metabolism and the utilization of alternative energy substrates to glucose could be beneficial [1,2]. In a recent clinical study performed on TBI patients, we identified valine, β-hydroxyisobutyrate (ibHB) and 2-ketoisovaleric acid (2-KIV) as three of the main predictors of TBI outcome [2]. In particular, we reported a significant association between cerebral microdialysis (CMD) ibHB, CMD 2-KIV (the precursor of ibHB) and CMD valine with short-term outcome (therapy intensity level (TIL)) [2]. Interestingly, another clinical study reported lower levels of valine in plasma of TBI patients [3] and, in a mouse model of TBI, the concentration of valine in the hippocampus was reduced to half as compared to control [4], suggesting that the level of the metabolite might be related to the pathology.

Valine is transported to the brain notably through facilitated transporters [5,6], and there is evidence that it is also locally released in the brain [7,8]. Interestingly, it was shown that rat primary astrocytes can produce large amounts of ibHB and 2-KIV from valine [9,10], and that both ibHB and 2-KIV are transported through the monocarboxylate transporters 1 (MCT1) [11] expressed at the neurovascular unit [12], suggesting transport through the blood–brain barrier and neurons. Although all types of neural cells can potentially convert valine to ibHB [9], astrocytes are of particular interest due to their pivotal role in metabolism and in supporting brain function [13]. ibHB and 2-KIV can be used as alternative energy substrates and provide new carbon skeletons into the TCA cycle (i.e., new synthesis of glutamate/GABA molecules), contributing therefore to the anaplerotic function of the astrocytes [9,10]. Moreover, the liver is the main organ for nutrient metabolism following oral intake or intravenous supplementation [14]. In particular, branched-chain amino acids (BCAAs) are metabolized in the liver [15] and the resulting products can be further used in multiple metabolic processes, such as energy production, urea metabolism and protein synthesis [16]. Given our previous findings on the association of ibHB and 2-KIV with TBI clinical outcome [2], understanding the metabolism of these key metabolites—beyond valine—and their delivery to the brain has therefore potential therapeutic benefits.

In the present study, we demonstrated the correlation between circulating and brain metabolites identified as predictive markers in TBI patients [2], suggesting a potential avenue to increase the level of ibHB and 2-KIV via nutritional intervention. Based on these data, we speculated that the liver can metabolize valine and provide significant amounts of intermediate metabolites, which can be further metabolized in the brain. To explore this further, we assessed the secretion of ibHB and 2-KIV in human liver hepatocellular cells (HepG2) and in human induced pluripotent stem cell-derived (iPSC) astrocytes, which play a key role in brain energy metabolism, using [U-^13^C]-valine. Overall, the findings suggest that both ibHB and 2-KIV are produced from valine to a large extent in both cell types. This could be of interest for the design of optimal nutritional interventions aiming at increasing the levels of ibHB and 2-KIV in the brain of TBI patients.

## 2. Materials and Methods

### 2.1. Patient Cohort and Sample Collection

Twelve patients with severe TBI were admitted to the Department of Intensive Care Medicine (ICU), at the Centre Hospitalier Universtaire Vaudois (CHUV)—Lausanne University Hospital, in Lausanne, Switzerland (9 males and 3 females, 56.7 ± 18.1 years old), as described previously [2]. Patients underwent brain monitoring with CMD probes as part of standard patient care. Approval for this study was obtained from the Ethical Committee of the University of Lausanne (protocol 2016-00126, accepted 23 March 2016), and informed consent was obtained from each patient’s next of kin. Moreover, this study was in compliance with STROBE guidelines for reporting observational studies.

CMD collection consisted of an intraparenchymal (subcortical white matter, visually normal brain) catheter (20 kDa cut-off CMA 70^®^, CMAMicrodialysis AB, Solna, Sweden), which was constantly perfused (rate: 0.3 μL/min) with a sterile solution mimicking cerebrospinal fluid content through a pump (CMA 106^®^, CMAMicrodialysis AB), as described previously [17]. Samples were collected twice a day (every 12h) for a maximum of 7 days, representing 128 samples in total. Samples were frozen in liquid nitrogen and kept at −80 °C until liquid chromatography–mass spectrometry (LC–MS) analysis.

Blood (approximately 3 mL) was collected in EDTA tubes twice a day and centrifuged for 10 min at 2000× *g* at 4 °C. Then, plasma was stored in Eppendorf tubes and kept at −80 °C under further analysis.

### 2.2. Cell Culture

All experiments were approved by the Swiss Ethics Committees on Research Involving Humans. HepG2 cells were obtained from Sigma-Aldrich (Buchs, Switzerland). The cells (passage number 7 and 8) were cultured in DMEM low glucose (5 mM) (ref. 21885108, Thermo Scientific-Life Technologies, Zug, Switzerland) supplemented with 10% fetal bovine serum, 1% non-essential amino acids and 1% penicillin/streptamin, and kept in a humidified atmosphere (5% CO_2_) at 37 °C. The medium was changed every 2 days until experiment.

Differentiated human iPSCs (iCell astrocytes) were obtained from Cellular Dynamics International (CDI, Madison, WI, USA). The cells were thawed according to the manufacturer’s instruction. iPSC-derived astrocytes were cultured in high glucose (25 mM), pyruvate (1 mM)- and glutamine (4 mM)-containing DMEM (Thermo Scientific-Life Technologies, ref. 41966029) supplemented with 10% fetal calf serum and N2 complement. Cells were kept in culture in a humidified atmosphere (5% CO_2_) at 37 °C for 7 days with the medium changed at days 3 and 6.

### 2.3. Chemicals and Reagents

LC–MS-grade acetonitrile, ethanol, and isopropanol were purchased from VWR Internationals (Leuven, Belgium) and Merck (Darmstadt, Germany). Water was purified in house using a Milli-Q Advantage A10 system from Merck Millipore (Billerica, MA, USA). Acetic acid was supplied by Sigma-Aldrich (St-Louis, MO, USA). Pure chemicals used for internal and external calibration were purchased from Sigma-Aldrich (St-Louis, MO, USA), Larodan (Solna, Sweden), Toronto Research Chemical (Toronto, ON, Canada), CDN Isotopes Inc. (Pointe-Claire, QC, Canada), and Cambridge Isotopes Laboratories (Tewksbury, MA, USA). ^2^H- and ^13^C-labeled compounds were obtained from Sigma-Aldrich (St-Louis, MO, USA), Larodan Fine Chemicals AB (Malmoe, Sweden), Toronto Research Chemicals (Toronto, ON, Canada), Cambridge Isotope Laboratories Inc. (Tewksbury, MA, USA), and CDN Isotopes (Pointe Claire, QC, Canada) and used as internal standards. Perfusion fluid CNS was bought from Harvard Apparatus (Holliston, MA, USA) and used as a blank matrix for the human samples. The uniformly labeled tracer ^13^C_5_-valine (>99% ^13^C, [U-^13^C]-valine) was purchased from Cambridge Isotopes Laboratories (Tewksbury, MA, USA). Stocks of [U-^13^C]-valine was prepared in PBS at 266 mM with 0.3% PBS final concentration.

### 2.4. Preparation of Internal and External Standards

Individual 100 μM stock solutions of each internal standard were prepared from the corresponding commercially available powder and stored at −20 °C. On the day of analysis, a 0.1–0.5 μM internal standards mixture in mobile phase A was prepared and used for calibration standards and samples preparation.

External standards for CMD sample analysis were prepared as follows: individual 25–100 mM stock solutions of each analyte were prepared from the corresponding commercially available powder and stored at −20 °C. Then, a mixture of all analytes (concentration 0.06–1 mM) in methanol/water (1:1) was prepared. Different volumes of the analytes and internal standards solutions were then mixed, and the volume of each standard was manually adjusted to 0.5 mL with mobile phase A. A series of 8 calibration standards was obtained and further diluted by mixing 15 μL of each solution with 15 μL of perfusion fluid CNS in a PCR plate. The plate was sealed, placed in a Thermomixer Comfort C maintained at 4 °C and shaken for 5 min at 1000 rpm. After this mixing step, the calibration standards (approximate concentrations of 0.005–100 μM) were placed in the autosampler and analyzed.

### 2.5. Samples Preparation

Regarding human samples, CMD samples from consecutive time points were pooled until a volume of 15 μL was obtained. A volume of 15 μL of the sample was pipetted and mixed with 15 μL of dilution solution (0.1–0.5 μM internal standards solution in mobile phase A) in a PCR plate. The plate was sealed, placed in a Thermomixer Comfort C (Eppendorf AG, Hamburg, Germany) maintained at 4 °C and shaken for 5 min at 1000 rpm. The plate was placed in the autosampler and samples were immediately analyzed.

Cells were washed 3 times and incubated in serum-, valine-, and glutamine-free DMEM (Thermo Scientific-Life Technologies) containing 1 mM glucose and 800 µM [U-^13^C]-valine or PBS for up to 6 h (1 mL/well). N2 supplement was added in the iPSC-derived astrocyte experiment. Experiments were run in duplicates in three independent experiments (*n* = 3). Cell supernatant was collected at different time points (0, 5, 10, 30, 60, 120, 240 and 360 min) and stored at −80 °C until analysis. On analysis day, 25 µL of cell culture supernatant was mixed with 25 µL of dilution solution (50 µL internal standard stock solution diluted with 450 µL mobile phase A) in a PCR plate. The plate was sealed, placed in a Thermomixer Comfort C maintained at 4 °C and shaken for 5 min at 1000 rpm. Samples were placed in the autosampler and immediately analyzed. Cell monolayers were collected at different time points (0, 5, 10, 30, 60, 120, 240 and 360 min). At each time point, cells were immediately placed on ice to stop metabolism, washed with cold PBS and frozen. After collection, cell pellets were extracted once with 500 µL and once with 700 µL ethanol/water (7:3) followed by centrifugation at 17,500× *g* for 20 min. Supernatants were dried under vacuum at room temperature and the solid residues were reconstituted in 30 µL of mobile phase A and immediately analyzed. The pellets were stored at −80 °C until protein content quantification.

### 2.6. Liquid Chromatographic Separation and Mass Spectrometric Detection

Liquid chromatography was performed on a I-Class UPLC system (Waters Corporation, Milford, MA, USA) combining a binary pump, a FTN autosampler and a column oven. Chromatographic separation was achieved on a Waters ACQUITY UPLC BEH C8 Column (100 × 2.1 mm, 1.7 µm) with a binary solvent system at a flow rate of 450 µL/min. Mobile phase A was 0.1% acetic acid in water and B was 0.1% acetic acid in acetonitrile/isopropanol (1:1). The binary solvent gradient was as follows: 0.0–1.0 min at 0% B, 1.0–6.5 min from 0% to 100% B, 6.5–8.5 min 100% B, followed by 2 min of equilibration at initial conditions. Column oven temperature was set to 55 °C and the autosampler injection volume to 1 µL.

High-resolution mass spectrometric analyses of cell samples and CMD samples were performed on Q Exactive and Q Exactive plus mass spectrometers (both from ThermoFisher Scientific, Bremen, Germany), respectively. For CMD samples, detection was performed in the data-dependent mode (top 3) in the negative and positive ionization modes in two separate injections. Instrument parameters were identical for both ionization modes and were as follows: for MS^1^, mass range *m/z* 65–600, resolving power of 35,000 (at *m/z* = 200), automatic gain control (AGC) target 5 × 10^6^ and maximum injection time 120 ms. For MS^2^, resolving power of 17,500 (at *m/z* = 200), AGC target 1 × 10^5^, maximum injection time 50 ms, isolation window 2 Da, normalized collision energy (NCE) 40, intensity threshold 3 × 10^5^, and dynamic exclusion 5 s. The mass spectrometer was interfaced to the UPLC system using a HESI probe. The spray voltage was set to −4.3 or +4 kV depending on the ionization mode. For both the positive and negative ionization modes, the heater and capillary temperatures were set to 350 °C, sheath gas flow rate to 45 arbitrary units (AU), auxiliary gas to 15 AU and sweep gas to 1 AU.

For cell samples, detection was performed in the negative ionization mode over the mass range *m/z* 65–600 with a resolving power of 70,000 (at *m/z* = 200). Data were acquired in the profile mode with an AGC target of 5 × 10^6^ ions and a maximum injection time of 250 ms. The mass spectrometer was interfaced to the UPLC system using a HESI probe. The spray voltage was set to −4kV. The heater and capillary temperatures were both set to 350 °C. Sheath gas and auxiliary gas flow rate were set to 45 and 15 AU, respectively. Instruments were calibrated every 4 days according to manufacturer specifications.

### 2.7. Data Analysis

Data analyses were performed with Xcalibur software 2.2 SP1 (ThermoFisher Scientific, Bremen, Germany). MS^1^ chromatograms were extracted using a mass tolerance of 5 ppm and signals were integrated with the ISIS algorithm.

Regarding cell data, absolute concentrations were calculated by multiplying the area ratio by the internal standard amount. Time-course data (cells and media) were subtracted to initial conditions (i.e., cells and media before incubation, respectively) and normalized to protein content. In CMD samples, analytes were quantified using external calibration curves.

Mass isotopomers differ by the number of ^13^C atoms in the molecule. C0 represents the fraction of unlabeled molecules in the total molecule pool, C1 the fraction of molecules labeled in one carbon, C2 in two carbons, etc.

### 2.8. Protein Content

Protein content was determined on cell pellets with a bicinchoninic acid-based protein assay (BCA protein assay kit 10678484; Thermo Scientific), with bovine serum albumin as the standard.

### 2.9. Glucose Quantification

The extracellular glucose concentration was determined using a glucose assay kit (Abnova kit KA0831).

### 2.10. Statistics

Data are shown as the mean ± SD. Normality testing was performed using the Shapiro–Wilk test, with statistical significance at *p* < 0.05. Correlations of not normally distributed data were quantified with Spearman rank correlation coefficients. Bonferroni correction was performed on the correlation data for reference. Human CMD and plasma data comparison was performed using the non-parametric Mann–Whitney test (unpaired), with statistical significance at *p* < 0.05. Time courses of in vitro data were followed up using paired t-test with statistical significance at *p* < 0.05. The AUC was assessed using one-way ANOVA with Tukey’s post-test for multiple comparisons.

## 3. Results

**Figure 1:**
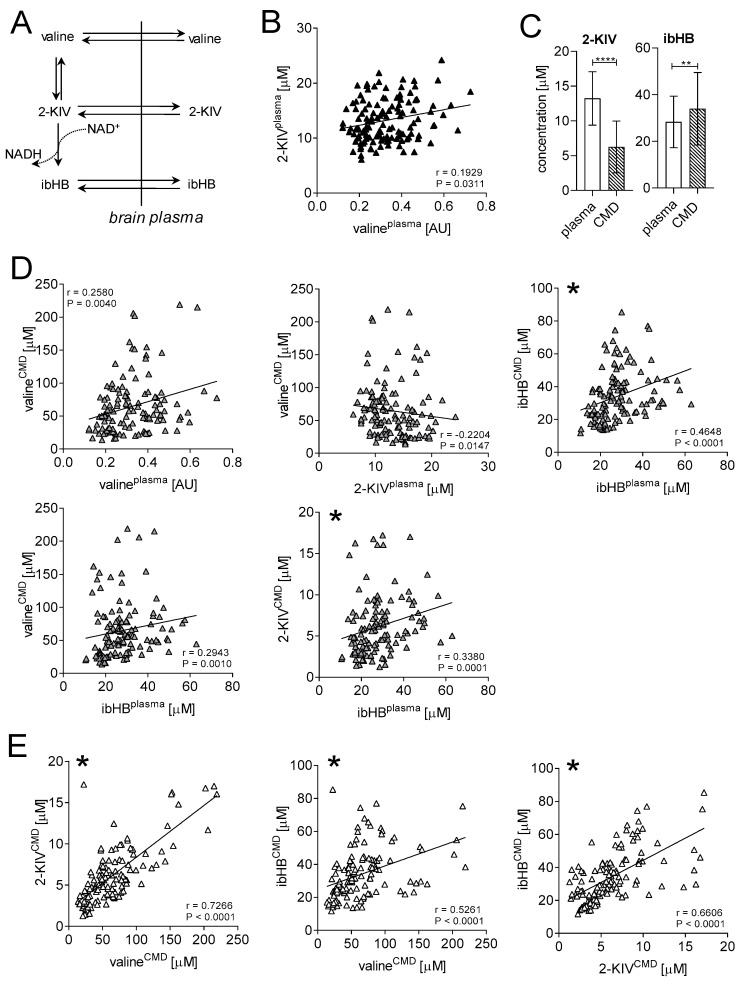
Correlation analysis of plasma and CMD metabolites identified previously as predictive markers of TBI outcome [2]. (**A**) Schematic overview of valine metabolism and transport of intermediates to the brain. (**B**) Plasma-specific Spearman correlation—plasma valine and 2-KIV. (**C**) Average concentrations of 2-KIV and ibHB measured in plasma and CMD. (**D**) Spearman correlations between plasma valine CMD valine, plasma 2-KIV and CMD valine, plasma ibHB and CMD ibHB, plasma ibHB and CMD valine, and plasma ibHB and CMD 2-KIV. (**E**) Brain-specific Spearman correlations—CMD valine and 2-KIV, CMD valine and ibHB, and CMD 2-KIV and ibHB. ** *p* < 0.01, and **** *p* < 0.0001. Mann–Whitney test. Graphs with * on the left corner are correlations that survived Bonferroni correction (α = 0.05/15 = 0.0033). AU: arbitrary unit. The line represents linear regression.

To determine the potential correlation between precursors and products of valine metabolism (Figure 1A) in both CMD and plasma, we performed Spearman correlations for related metabolites. We observed significant positive correlations between plasma valine and plasma 2-KIV (r = 0.1929; *p* = 0.0311; Figure 1B), plasma and CMD valine (r = 0.2580; *p* = 0.0040; Figure 1D), plasma and CMD ibHB (r = 0.4648; *p* < 0.0001; Figure 1D), plasma ibHB and CMD 2-KIV (r = 0.3380; *p* = 0.0001; Figure 1D) and plasma ibHB and CMD valine (r = 0.2943; *p* = 0.0010; Figure 1D). A higher level of ibHB was detected in CMD compared to plasma, while the opposite was true for 2-KIV (Figure 1C). A negative correlation was moreover found between plasma 2-KIV and CMD valine (r = −0.2204; *p* = 0.0147; Figure 1D). In addition, significant positive correlations were found in the brain between valine and 2-KIV (r = 0.7266; *p* < 0.0001; Figure 1E), valine and ibHB (r = 0.5261; *p* < 0.0001; Figure 1E), and 2-KIV and ibHB (r = 0.6606; *p* < 0.0001; Figure 1E). Supplementary Table S1 shows the Spearman coefficients for all correlations. It is important to note that only plasma-CMD ibHB and plasma ibHB-CMD 2-KIV survived Bonferroni correction (α = 0.05/15 = 0.0033).

**Figure 2:**
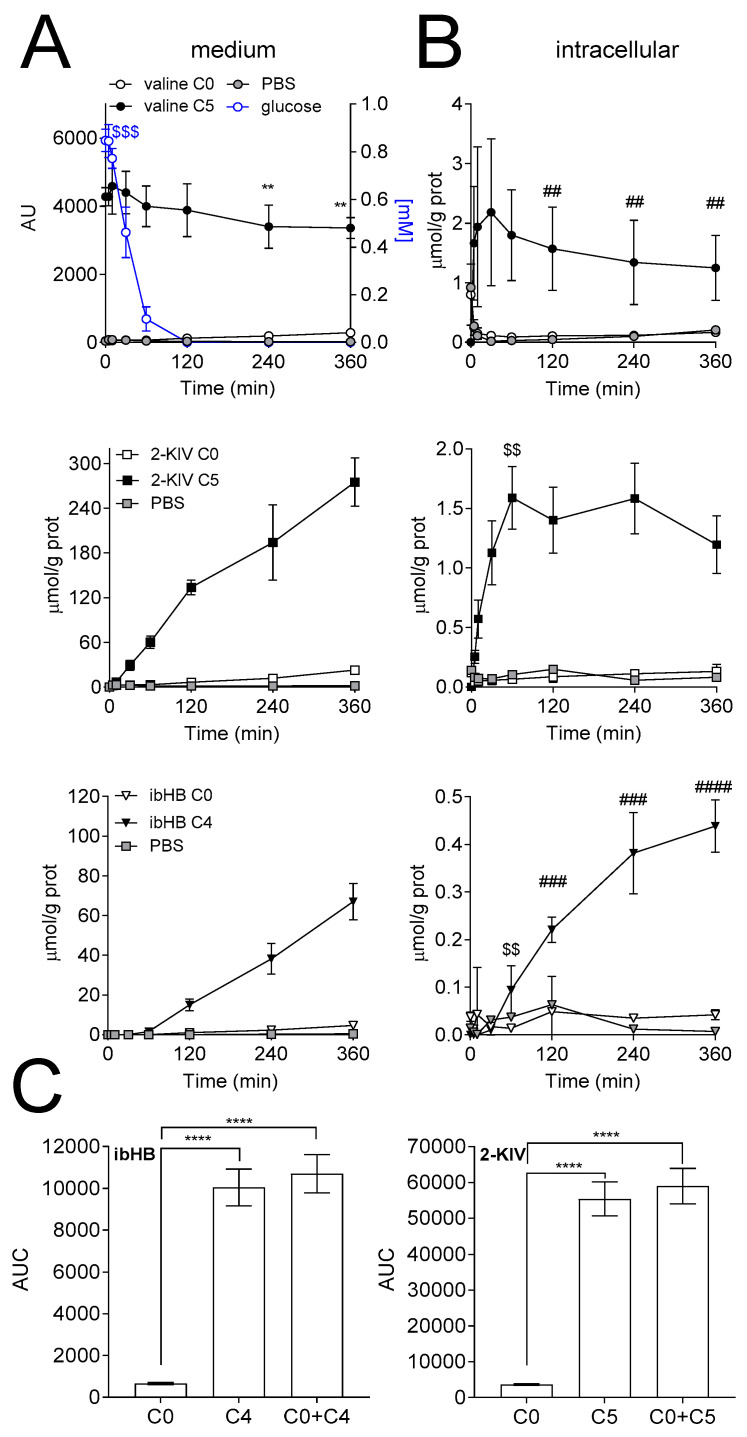
Isotopomer analysis in hepatocytes. Extracellular (**A**) and intracellular (**B**) time courses of valine, 2-KIV and ibHB concentrations in the HepG2 experiment. (**C**) AUC of the secreted ibHB and 2-KIV. Glucose concentration was measured in the medium and is depicted in blue (A). (*n* = 3). ** *p* < 0.01, as compared to 5 min; ## *p* < 0.01, ### *p* < 0.001, and #### *p* < 0.0001, as compared to 60 min; $$ *p* < 0.01 and $$$ *p* < 0.001, as compared to 30 min paired t-test. (C) **** *p* < 0.0001, one-way ANOVA with Tukey’s post-test for multiple comparisons. AU: arbitrary unit.

**Figure 3:**
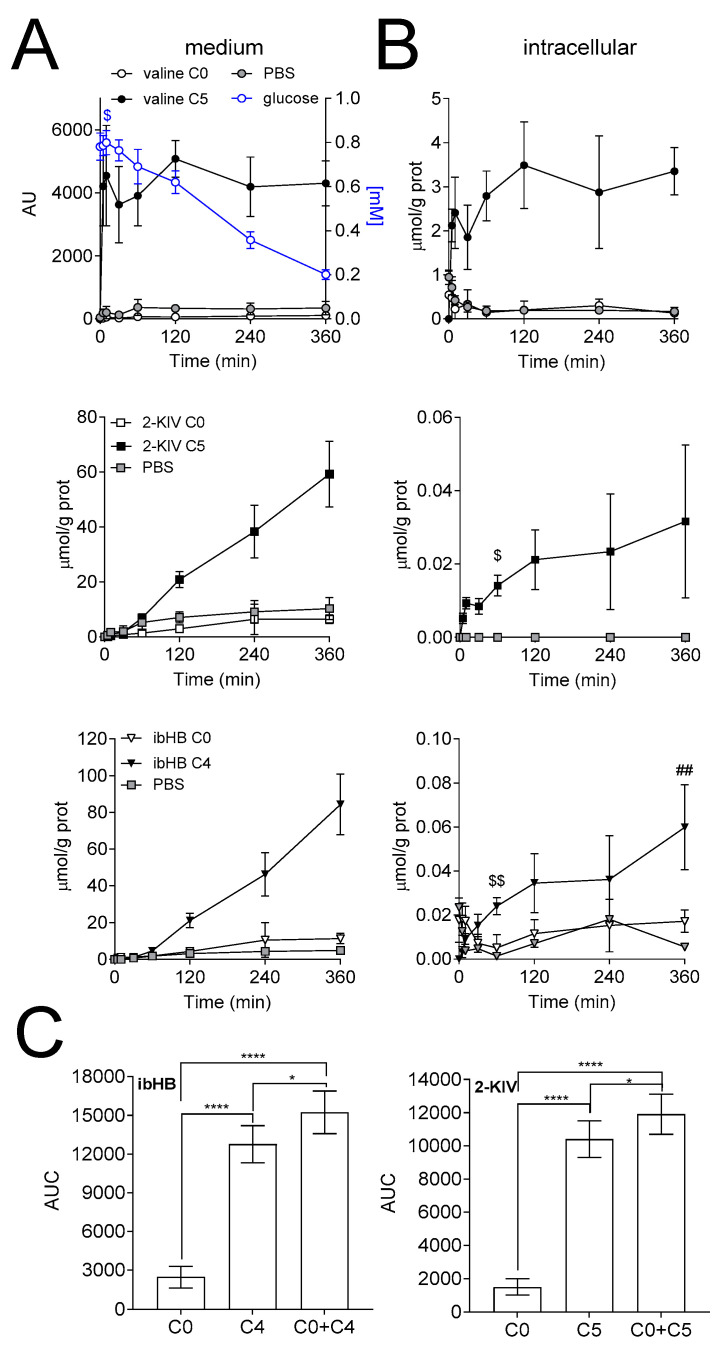
Isotopomer analysis in astrocytes. Extracellular (**A**) and intracellular (**B**) time courses of valine, 2-KIV and ibHB concentrations in the iPSC-derived astrocyte experiment. (**C**) AUC of the secreted ibHB and 2-KIV. Glucose concentration was measured in the medium and is depicted in blue (A). (n = 3). ## *p* < 0.01, as compared to 60 min; $ *p*<0.05 and $$ *p* < 0.01, as compared to 30 min paired t-test. (C) * *p* < 0.05 and **** *p* < 0.0001, one-way ANOVA with Tukey’s post-test for multiple comparisons. AU: arbitrary unit.

To determine the contribution of valine to the downstream metabolites, mass isotopomers of ibHB and 2-KIV were quantified overtime. Fully labeled and unlabeled intermediates of the valine metabolic pathway were detected in the medium and intracellular space of HepG2 cells and iPSC-derived astrocytes upon incubation with [U-^13^C]-valine. In general, significant larger production of total ibHB (unlabeled + fully labeled = C0 + C4) and 2-KIV (unlabeled + fully labeled = C0 + C5) were measured in the presence of valine, as compared to control (Figure 2 and Figure 3).

In HepG2 cells, the secretion of 2-KIV started after 5 min of incubation with valine, and the levels of measured 2-KIV C5 and 2-KIV C0 increased to 275.2 ± 32.6 and 22.8 ± 2.4 µmol/g prot, respectively, after 360 min. However, ibHB secretion started at 60 min following incubation with valine. After 360 min, 4.71 ± 0.60 µmol/g prot and 67.02 ± 9.13 µmol/g prot of ibHB C0 and C4 were detected in the medium. The production of these intermediates was accompanied by a significant decrease (*p* < 0.01) of valine C5 in the medium at 240 and 360 min (Figure 2A). The area under the curve (AUC) also significantly differed between ibHB C0 and C4, and 2-KIV C0 and C5 (*p* < 0.0001). In particular, the AUC was 656 ± 50, 10,041 ± 878 and 10,697 ± 919 for the C0, C4 and C0 + C4 labeling counterparts of ibHB, respectively, corresponding to a labeled/[unlabeled + labeled] ratio of 94 ± 12%. The AUC of 2-KIV were 3607 ± 244, 55,386 ± 4750 and 58,993 ± 4951 for the C0, C5 and C0 + C5 labeling counterparts of 2-KIV, respectively, corresponding also to a labeled/[unlabeled + labeled] ratio of 94 ± 12% (Figure 2C). 

The glucose concentration in the medium was 0.82 ± 0.9 mM during the first 10 min before decreasing to 0.10 ± 0.05 mM after 60 min of incubation with [U-^13^C]-valine. The medium was deprived from glucose after 120 min until the end of the experiment (Figure 2A).

Quantification of intracellular valine showed that the endogenous pool became labeled 5 min after the incubation onset. A significant (*p* < 0.01) decrease in valine concentration was measured at 120, 240 and 360 min as compared to 60 min (Figure 2B). Regarding the intermediates, the steady state was reached after 60 min for 2-KIV C5, while a small time-dependent increase was observed for ibHB C4 (Figure 2B).

Similar mass isotopomer analysis was performed in iPSC-derived astrocytes. The secretion of 2-KIV started after 5 min and increased to 59.3 ± 11.9 and 6.4 ± 1.6 µmol/g prot of 2-KIV C5 and 2-KIV C0, respectively after 360 min. Similarly, the secretion onset of ibHB started after 5 min of incubation and reached to 11.4 ± 2.9 µmol/g prot and 84.3 ± 16.6 µmol/g prot of ibHB C0 and C4 after 360 min (Figure 3A). A significant difference (*p* < 0.0001) between the AUC of secreted ibHB C0 and C4, and 2-KIV C0 and C5 was observed. In particular the AUC was 2478 ± 817, 12,759 ± 1436 and 15,238 ± 1653 for the C0, C4 and C0+C4 labeling counterparts of ibHB, respectively, corresponding to a labeled/[unlabeled + labeled] ratio of 84 ± 16% (Figure 3C). Regarding 2-KIV, the AUC was 3607 ± 244, 55,386 ± 4750 and 58,993 ± 4951 for the C0, C5 and C0 + C5 labeling counterparts of 2-KIV, respectively, corresponding to a labeled/[unlabeled + labeled] ratio of 87 ± 15%.

The glucose concentration in the medium was 0.79 ± 0.09 mM during the first 10 min and then linearly decreased over time. By the end of the experiment, the glucose concentration was 0.2 ± 0.02 mM (Figure 3A).

Quantification of intracellular metabolite demonstrated that the endogenous pool of valine became labeled 5 min after the incubation onset. ibHB C4 exhibited a small increase over time and 2-KIV C5 reached steady state after 60 min of incubation (Figure 3B).

## 4. Discussion

In a recent clinical study performed on TBI patients, we identified changes in valine, ibHB and 2-KIV in the CMD fluid as the main predictors of TBI outcome [2]. These two valine catabolites can potentially serve energy metabolism or anaplerosis [2,9,10]. 2-KIV might moreover be protective against oxidative stress [10]. Given therefore the pivotal role that they can have in the brain, we aimed to investigate the potential avenue to increase the level of ibHB and 2-KIV in the brain via valine supplementation.

HepG2 cells and iPSC-derived astrocytes were incubated with [U-^13^C]-valine or PBS (control). In general, the production of total ibHB (C0 + C4) and 2-KIV (C0 + C5) were significantly higher in the presence of valine as compared to control, suggesting substantial metabolism of valine in these cells. Noticeably, the main precursor of ibHB and 2-KIV was identified as valine: ibHB C4 and 2-KIV C5 in the HepG2 medium were 94 ± 12% of total ibHB (C0 + C4) and 94 ± 12% of total 2-KIV (C0 + C5), respectively, and in iPSC-derived astrocytes these ratios were 84 ± 16% and 87 ± 15% for total ibHB and total 2-KIV, respectively. These in vitro observations of valine metabolism in both HepG2 cells and iPSC-derived astrocytes confirm the associations found in the clinical data, namely the correlations between plasma valine and 2-KIV, CMD valine and 2-KIV, CMD valine and ibHB, and CMD 2-KIV and ibHB. Indeed, in addition to transport through the blood–brain barrier [5], we speculate that these positive correlations arise also from the conversion of valine to 2-KIV and ibHB in both the liver and the brain.

Overall iPSC-derived astrocytes produced more ibHB and less 2-KIV upon valine administration compared to HepG2 cells. This in vitro observation is in line with our observation in the clinical study, namely plasma 2-KIV level was higher than CMD 2-KIV, and CMD ibHB was higher than plasma ibHB. This suggests thus that ibHB might mainly be produced in the brain, while 2-KIV in the liver.

The secretion onset of ibHB was earlier in iPSC-derived astrocytes compared toHepG2 cells, suggesting faster release. While the secretion of 2-KIV started after 5 min in both HepG2 and iPSC-derived astrocytes, a significant time delay was observed regarding ibHB production in HepG2. Interestingly, the lag matches the decrease in the glucose concentration measured in the medium. After 1 h when the glucose concentration reached approximately 100 µM, ibHB C4 started to be produced. Thereafter, as glucose became relatively depleted in the medium, ibHB C4 secretion increased drastically. Although this requires further investigation, this observation might suggest that glucose uptake impairs ibHB but not 2-KIV production in HepG2 cells. The drastic decrease in extracellular glucose did not occur in iPSC-derived astrocytes and after 360 min of incubation, approximately 200 µM of glucose remained in the medium. In addition, the secretion of 2-KIV and ibHB started after 5 min of incubation in iPSC-derived astrocytes; the mechanism for ibHB production and secretion seems to be different in both investigated cells types.

Nonetheless, the fact that HepG2 cells substantially secrete valine intermediates suggest that the correlations between plasma and CMD valine, as well as plasma and CMD ibHB, might arise from the transport of these metabolites to the brain through the blood–brain barrier [5]. The larger concentrations of CMD ibHB than plasma ibHB indicate moreover active production of ibHB in the central nervous system. Interestingly, a negative correlation was found between plasma 2-KIV and CMD valine. While CMD valine was significantly correlated to CMD 2-KIV, no correlation was observed for plasma and CMD 2-KIV (data not shown). Furthermore, concentration of plasma 2-KIV was higher than concentration of CMD 2-KIV. Although further investigation is required, this suggests that other factors might contribute to plasma and CMD 2-KIV. Finally, as branched-chain 2-oxo acid dehydrogenase catalyzes irreversibly the conversion of 2-KIV into isobutyryl-CoA [9,10,18], the correlations between plasma ibHB and CMD valine/2-KIV might notably be due to the release of ibHB from the brain to the circulation. Indeed, ibHB and 2-KIV have been reported to be released from the muscle [19,20] to be further metabolized in the liver for gluconeogenesis [21]. ibHB could therefore play the role of an inter-organ metabolite [9].

The intracellular pools of 2-KIV C5 reached steady state after 60 min in both HepG2 and iPSC-derived astrocytes. Interestingly, it coincides with the time when the increase in intracellular ibHB C4 started. The conversion of 2-KIV into ibHB consumes NAD+ and produce NADH [10]. It was previously reported that 3-hydroxyisobutyrate dehydrogenase, converting ibHB into methylmalonic semialdehyde, is inhibited by NADH [22], suggesting that ibHB accumulation might result from an increase NADH/NAD^+^ ratio over time [22,23], eventually leading to its release from the cell [24]. This is in line with the small increase in intracellular ibHB observed in both HepG2 and iPSC-derived astrocytes. Finally, the decrease in intracellular valine in HepG2 from 120 min could be explained by increase in valine catabolism and decrease in extracellular valine level. Murin et al. showed a decrease in extracellular valine in primary astrocytes after 12 h of incubation [10]. Although longer experiments is required to further assess changes in extracellular valine—not only in iPSC-derived astrocytes but also under reactive astrogliosis that characterizes brain injury [25]—these data further point to the liver as a main organ for valine metabolism, and therefore suggests valine as a good candidate for nutritional intervention to increase levels of related metabolites in the brain.

## 5. Conclusions

Overall, these findings suggest that although there are differences in secretion onsets and total amounts, ibHB and 2-KIV are produced largely from valine in both the liver and astrocytes. This could be of interest in the design of optimal nutritional interventions aiming at increasing the levels of not only valine, but also ibHB and 2-KIV in the brain. BCAA supplementations, and in particular valine supplementation, have already been assessed both in animal models of TBI and TBI patients. Oral administration of a BCAA mixture, containing 100 mM of valine, for 5 days in a mouse model of TBI restored the concentrations of BCAAs in the hippocampus and synaptic efficacy, leading to an improvement in cognitive performance [4]. TBI patients receiving a BCAA solution, 46% of which was valine, for 15 days intravenously significantly improved plasma valine concentrations and cognitive recovery [3]. Together with the present results, valine supplementation could thus be a promising approach for TBI rehabilitation, as altering ketometabolism by valine supplementation could potentially change the metabolic states of the patients and therefore improve the outcome.

## Supplementary Materials

The following are available online at https://www.mdpi.com/2227-9059/8/11/487/s1, Table S1: Spearman correlation coefficient for all possible comparisons of CMD and plasma data.
