# Supplementary material for: Exploring Valine Metabolism in Astrocytic and Liver Cells: Lesson from Clinical Observation in TBI Patients for Nutritional Intervention
Source: Biomedicines. 2020 Nov 10;8(11):487. doi: 10.3390/biomedicines8110487 (PMC7697144; doi:10.3390/biomedicines8110487)
Supplement: Supplementary file 1 [file biomedicines-08-00487-s001.pdf]

Assessing valine metabolism in brain and liver cellular models: Lesson from clinical observation in TBI patients for nutritional intervention

Sarah Sonnay, Nicolas Christinat, Jonathan Thevenet, Andreas Wiederkehr, Anirikh Chakrabarti, Mojgan Masoodi

plasma

		valine	2-KIV	ibHB
plasma	valine			
	2-KIV	0,1929*		
	ibHB	0,1387	-0,1653	

plasma

		valine	2-KIV	ibHB
CMD	valine	0,2580*	-0,2204*	0,2943***
	2-KIV	0,1615	-0,1006	**0,3380*** **
	ibHB	0,01333	-0,1078	**0,4648*******

CMD

		valine	2-KIV	ibHB
CMD	valine			
	2-KIV	**0,7266*******		
	ibHB	**0,5261*******	**0,6606*******	

Supplementary table S1 : Spearman correlation coefficient for all possible comparisons of CMD and plasma data.

* p < 0.5, *** p < 0.001, **** p < 0.0001. Coefficients in bold survived Bonferroni correction (α = 0.05/15 = 0.0033).
